# Genetic Removal of the CH1 Exon Enables the Production of Heavy Chain-Only IgG in Mice

**DOI:** 10.3389/fimmu.2018.02202

**Published:** 2018-09-25

**Authors:** Tianyi Zhang, Xueqian Cheng, Di Yu, Fuyu Lin, Ning Hou, Xuan Cheng, Shanshan Hao, Jingjing Wei, Li Ma, Yanbin Fu, Yonghe Ma, Liming Ren, Haitang Han, Shuyang Yu, Xiao Yang, Yaofeng Zhao

**Affiliations:** ^1^State Key Laboratory of Agrobiotechnology, College of Biological Sciences, National Engineering Laboratory for Animal Breeding, China Agricultural University, Beijing, China; ^2^State Key Laboratory of Proteomics, Beijing Proteome Research Center, National Center for Protein Sciences (Beijing), Beijing Institute of Lifeomics, Beijing, China

**Keywords:** HcAbs, nanobody, CH1 domain, mouse, phage display, single domain antibodies

## Abstract

Nano-antibodies possess great potential in many applications. However, they are naturally derived from heavy chain-only antibodies (HcAbs), which lack light chains and the CH1 domain, and are only found in camelids and sharks. In this study, we investigated whether the precise genetic removal of the CH1 exon of the γ1 gene enabled the production of a functional heavy chain-only IgG1 in mice. IgG1 heavy chain dimers lacking associated light chains were detected in the sera of the genetically modified mice. However, the genetic modification led to decreased expression of IgG1 but increased expression of other IgG subclasses. The genetically modified mice showed a weaker immune response to specific antigens compared with wild type mice. Using a phage-display approach, antigen-specific, single domain VH antibodies could be screened from the mice but exhibited much weaker antigen binding affinity than the conventional monoclonal antibodies. Although the strategy was only partially successful, this study confirms the feasibility of producing desirable nano-bodies with appropriate genetic modifications in mice.

## Introduction

Conventional antibodies are hetero-tetrameric proteins consisting of two identical heavy chains and two identical light chains connected by disulphide bonds (1, 2). Each antigen binding site is thus formed by the variable regions of both heavy and light chains. However, heavy chain-only antibodies (HcAbs), which are found naturally in camelids and sharks, comprise only two heavy chains (3–5). Despite the absence of light chains in HcAbs, the heavy chain variable region itself binds antigens normally (6–8). The variable region of either camelid HcAbs (called VHH) or the shark IgNAR heavy chain (called vNAR) is called the nano-antibody or single domain antibody (SdAb) (9–11). Since their discovery more than 25 years ago, nano-antibodies have been used for research purposes, small chemical analyses, clinical diagnosis, and therapeutic applications (1, 12–16), due to their apparent advantageous characteristics, such as a small size and better solubility and stability than conventional antibodies (10, 17). More importantly, with their longer CDR3 (complementarity determining region 3) (18, 19), VHH, or vNAR can recognize recessed cryptic epitopes or active sites of enzymes that are not bound by conventional antibodies (20–22). On this topic, an excellent summary was recently given in a review paper by Smider et al. (23).

An immunized VHH cDNA library is usually needed for screening to obtain a desirable VHH for a particular target (24). One preliminary consideration for the construction of an immunized VHH library is the immunization of camelids (25, 26), such as alpacas or camels, which appears to be a practical problem for most laboratories studying VHH antibodies. The management of these large animals clearly requires specific knowledge of animal husbandry and is more difficult and expensive than handling laboratory animals such as mice, rats, or rabbits. As different animal individuals usually respond differently to immunization with certain antigens (27), a large number of animals may sometimes be needed to guarantee that a VHH with a desirable affinity for a specific antigen is successfully obtained, thus further increasing the cost and difficulty for many laboratories working with these large animals.

Despite the superior advantages of the camelid VHH compared with conventional antibodies (6), the abovementioned issues have encouraged us to examine the possibility of producing HcAbs or nano-antibodies using genetically modified laboratory animals such as mice. Structurally, HcAbs differ from conventional antibodies by the absence of light chains and the CH1 domain in the heavy chain constant region (19), which usually covalently binds to light chains (28). More essentially, as compensation for the lack of light chains, VHHs have been endowed with some special amino acid changes encoded by germline VHH gene segments in the camelid genome and a longer CDR3 that confers improved solubility and thermal stability and the ability to bind unusual epitopes (20, 29). Thus, the following two issues must be addressed before mice are genetically modified to produce functional HcAbs: first, whether the genetic removal of CH1 exon enables the production of heavy chain-only antibodies, and second, whether a VH that is functionally similar to camelid VHH is generated in these genetically modified mice *in vivo* following immunization (19).

The natural absence of the CH1 domain in camelid HcAbs results from a point mutation (G > A) in the 5′ splice site of the intron between the CH1- and hinge-encoding exons, leading to skipping of the CH1 exon during RNA splicing (30, 31). The lack of CH1 also accounts for the secretion of HcAbs by camelid B cells, as typically, prior to assembly with light chains into conventional antibodies, the CH1 domain would be bound by the chaperonin protein BiP (GRP78), which prevents the secretion of the heavy chain (32–34). The first question concerning whether genetic removal of the CH1 exon enables the production of HcAbs must be answered to produce functional single VH domain antibodies in genetically modified mice. Evidence derived from light chain-deficient mice or chickens supports the feasibility of this approach, as some spontaneous HcAbs have been detected in these animals (35, 36). These spontaneous HcAbs likely resulted from abnormal class switch recombination, deleting the CH1 exon of a certain Ig class-encoding gene.

Based on available data, HcAbs are highly likely to be expressed following genetic removal of the CH1 exon, but the key question remains of whether the expressed HcAbs are actually functional and whether these expressed HcAbs can be used to generate nano-antibodies with a high affinity for particular antigens that is comparable to natural camelid VHHs. Compared with ordinary VH, camelid VHHs feature specific amino acid residues in FR2 (framework region 2) and a longer CDR3 that usually contains an additional loop formed by disulfide bonds between non-canonical cysteines (37–40). Although typical VHH sequences are not encoded in the mouse immunoglobulin heavy chain gene locus, we speculate that extensive VDJ recombinations and somatic hypermutations may be sufficient to create VHs that are structurally similar to camelid VHHs *in vivo*. Based on the aforementioned hypothesis, we examined whether mice are a useful model to generate functional nano-antibodies in the present study by genetically deleting the γ1 CH1 exon from the mouse genome.

## Materials and methods

### Targeting vector construction and generation of mice

The position of Cγ1 was identified based on the mouse IgG1 gene sequence from the NCBI website (accession number: D78344). We chose sequences located 2671 bp upstream (at the 5′-end of the *Cla* I site and at the 3′-end of the *Sal* I site) of the γ1 CH1-encoding exon as the short homologous arm and sequences located 4,725 bp (From *Not* I to *Xho* I site) downstream that included the γ1 Hinge- to the M2-encoding exon as the long homologous arm. Target sequences were amplified using KOD polymerase (Toyobo, Kita-ku, Osaka, Japan) from the 129Sv/J genome. Two fragments then were assembled into the pPN III targeting vector, which contained two *lox*P sites in the same direction flanking the *neo* gene.

The targeting vector was subsequently transfected into ES cells from 129Sv/J mice using standard protocols. After positive selection of *neo* and negative selection of TK, we then screened the positive ES cell clones with short arm- and long arm-specific primers using LA-Taq (TaKaRa, Kusatsu, Shiga, Japan) and the following programme: 94°C for 5 min, followed by 35 cycles of 94°C for 50 s, 62°C/65°C for 30 s, and 72°C for 5 min, and then a final extension step at 72°C for 7 min. The *neo* gene was amplified using DIG-labeled probes to confirm the positive clones, and the pPN III vector, genomic DNA from wild type 129Sv/J mice and negative ES cells were used as controls. Hybridization and detection were performed using the DIG High Prime DNA Labeling and Detection Starter Kit II (Roche, Basel, Switzerland), according to the manufacturer's instructions. Positive ES cell clones were subsequently microinjected into the blastula of C57BL/6J mouse using standard protocols.

Genomic DNA was isolated from tail biopsies of F0 generation mice via phenol/chloroform extraction, and homologous arms were confirmed by PCR using LA-Taq. As the F0 generation mice were chimeras, they were then crossbred with C57BL/6J WT mice to obtain heterozygotes. The offspring homozygous mice were screened by PCR and Southern blotting using the same methods applied to the F0 generation mice.

Tg (EIIa-cre) C5379 Lmgd mice (129Sv/J) were provided by Dr. Yang Fuxiao (Academy of Military Medical Sciences) and mated with the aforementioned mice to remove the *neo* gene. The *neo* gene was deleted using the Cre-*lox*P system. We then obtained our target HG1 mice, which were also confirmed by PCR and Southern blotting.

All WT mice were purchased from Beijing Vital River Laboratory Animal Technology Co., Ltd. Mice were housed in individually ventilated cages at an ambient temperature of 21–23°C with an automated 12:12 h light-dark cycle and access to water and commercial rodent food. Animal care was administered in accordance with the guidelines of China Agricultural University for animal welfare. All animal experiments performed in the present study were approved by the Animal Care and Use Committee of China Agricultural University.

### RNA isolation, RT-PCR, qPCR, and 5′ RACE

Total RNA was isolated from the spleens of 8- to 10-week-old positive mice using an RNeasy Mini Kit (QIAGEN, Dusseldorf, Germany), and the RNA concentration was measured with Nanodrop 2000 (Thermo Fisher Scientific, Rockford, IL, USA). Reverse transcription was performed using M-MLV Reverse Transcriptase and oligo(dT) 20 primers according to the manufacturer's instructions (Promega, Madison, WI, USA). RT-PCR was used to analyse the transcription of truncated IgG1 with specific JH forward and γ1-CH2 reverse primers. Mouse GAPDH was amplified as an internal control. qRT-PCR was performed using LightCycler 480 SYBR Green I Master mix (Roche, Basel, Switzerland) with primers for IgG1, IgM and GAPDH under the following cycling conditions: 95°C for 5 min, followed by 40 cycles of 95°C for 10 s, 60°C for 10 s, and 72°C for 10 s. The relative transcription levels of IgG1 and IgM were determined using the 2^−ΔΔCt^ method by comparing the values with the internal control GAPDH.

Furthermore, the recombined variable region sequences of the truncated IgG1 or IgM were amplified by 5′ RACE according to the manufacturer's protocol (Invitrogen, BioSource International, USA). All variable region sequences were blasted against the IMGT database (http://www.imgt.org/ligmdb/) to analyse the V, D, J, and CDR3 sequences.

### Serum treatment and western blotting

Collected serum samples were treated with protein L magnetic beads (Thermo Fisher Scientific, Rockford, IL, USA) according to the manufacturer's protocol to obtain serum samples containing light chain-free immunoglobulin. The serum samples collected from 8- to 12-week-old HG1 mice were separated by SDS-PAGE, electrophoretically transferred onto Immobilon-P membranes (Millipore, Burlington, MA, USA) and treated using a standard protocol. Immunodetection was conducted with HRP-conjugated antibodies, including goat anti-mouse IgM (μ chain) (Rockland Immunochemicals, Limerick, PA, USA) and goat anti-mouse IgG1 heavy chain (Abcam, Cambridge, MA, USA). Enhanced chemiluminescence and autoradiography were performed using ECL Western blotting reagents (Amersham Biosciences, Amersham, Buckinghamshire, UK). Serum samples from age-matched WT mice served as negative controls.

### OVA immunization and ELISA

The OVA antigen (ovalbumin) was resuspended in PBS. Five 6- to 10-week-old HG1 or WT mice were immunized four times with 100 μg OVA at 2-week intervals. For the first immunization, the adjuvant was CFA (Sigma-Aldrich, St. Louis, MO, USA), the mice were immunized by subcutaneous injection, while subsequent immunizations were immunized by intraperitoneal injection utilized FA (Sigma-Aldrich, St. Louis, MO, USA). Serum samples were collected before the first immunization and 3–5 days after the last immunization. All immunoglobulin subtypes were handled according to the manufacturer's instructions (Bethyl, Montgomery, TX, USA), while OVA-specific antibodies were examined using the mouse anti-OVA ELISA kit according to the manufacturer's instructions (ADI, San Antonio, TX, USA). Serum samples were diluted to an appropriate concentration after the pre-test. Samples were assayed in triplicate for each measurement.

### Generation and panning of the phage-display library

RNA was extracted from the spleens of immunized mice after the last immunization. Reverse transcription was performed as mentioned above. Spleen cDNAs from HG1 mice were mixed for IgG1 variable region repertoire amplification. The VH segments of IgG1 from HG1 mice were amplified by nested PCR reactions as described below. In the first-round PCR, the recombined variable region from the leader sequence to the γ1-CH2-encoding exon was amplified using 2 × Taq PCR StarMix (GeneStar, Beijing, China). The amplification conditions comprised an initial denaturation step at 95°C for 5 min, followed by 25 PCR cycles of 95°C for 30 s, 53°C for 30 s, and 72°C for 1 min, and a final extension step of 72°C for 10 min. Approximately 700 bp fragments corresponding to the first-round PCR products were purified from the gel with a Qiaquick Gel Extraction Kit (QIAGEN, Dusseldorf, Germany). In the second-round PCR, equal amounts of amplified VH PCR products were mixed as the PCR template and amplified from the start of the recombined V gene to the J segment end under the same conditions as applied for the first-round PCR. The second-round PCR primers were modified to include a *Nco* I site upstream and a *Not* I site downstream of the target sequence to facilitate cloning into the display vector. Subsequently, equal amounts of the amplified VH fragments were mixed and digested with *Nco* I and *Not* I (NEB, Ipswich, MA, USA), and the ~400 bp VH products were constructed into the phage-display vector pHEN2 to generate a VH gene library with T4 ligase (NEB, Ipswich, MA, USA). Plasmids were transformed into *E. coli* strain TG1 by electroporation and grown at 37°C on 2YT culture plates supplemented with 100 μg/ml ampicillin and 2% glucose (2YTAG medium). TG1 were used to produce and select phages.

The transformed bacterial clones were titrated on agar plates to determine the library size, and colony PCR was performed to determine the presence of DNA inserts. The phage library containing 2 × 10^8^ clones was used for affinity selection. Glycerol stocks of the VH library were grown to log phase, rescued with M13 helper phages (NEB, Ipswich, MA, USA) and then amplified at 30°C overnight in 2YT medium supplemented with 100 μg/ml ampicillin and 50 μg/ml kanamycin. The phage was subsequently precipitated with PEG–NaCl (4% PEG and 0.5 M NaCl) and then resuspended in PBS.

After precipitation of the VH phage library, multiple rounds of affinity panning were performed to obtain the antigen-specific VH fragments. Antigens were coated on microtiter plates at three concentrations (80, 60, and 40 μg/well) overnight for each panning round, followed by an incubation with 2% skimmed milk-PBS solution as blocking buffer for 1 h at 37°C. The VH phage library was then incubated in wells that had not been coated with antigen for 1 h at room temperature to eliminate the background. Then, the background-removed VH phage library was incubated with the preblocked OVA-specific wells at room temperature for 1 h. Subsequently, the wells were washed with 0.1–0.3% phosphate-buffered saline/0.1% Tween 20 (PBST) to remove unbound phage. As the library was assumed to contain only phages that recognize OVA, a general trypsin (1 mg/ml) elution method was used to harvest the bound phages. The trypsin solution was then neutralized with 4% skimmed milk-PBS for at least 30 min. The selected phage clones were subsequently precipitated with PEG–NaCl (4% PEG and 0.5 M NaCl) and then resuspended in PBS and rescued with M13 helper phages.

### Phage ELISA

Following 3 rounds of the panning process, OVA-specific phage clones were obtained and a phage ELISA was used to examine the binding affinity. The microtiter plates were coated with 20 μg of OVA solution (~100 μl) per well, blocked with a 2% skimmed milk-PBS solution, and then phage clones were added and the plates were incubated at room temperature for 1 h. The plates were then washed five times with 0.1% PBST and finally incubated with an HRP-conjugated anti-M13 monoclonal antibody (Abcam, Cambridge, MA, USA). Subsequently, the plates were washed again with PBST and then incubated with the TMB peroxidase substrate (BioLegend, San Diego, CA, USA). The absorbance was then measured at 450 nm.

### Prokaryotic expression and purification

The selected VH fragments were individually amplified and digested with *Nco* I and *Not* I, subsequently ligated into PET-28a (+) plasmids with T4 ligase to generate prokaryotic expression vectors. A His6-tag was introduced at the C-terminus of the VH gene. Expression vectors were then transformed into *E. coli* strain BL21. *E. coli* were stimulated with 2 mM IPTG at 20°C overnight to induce the production of VH antibodies, which were then purified on Ni-NTA His•Bind resin (Merck, Kenilworth, NJ, USA) according to the manufacturer's protocol. The purified VH antibodies were dissolved in PBS for further detection.

### Protein-binding assays

The selected purified VH antibodies containing the His6-tag were screened for binding to OVA (Sigma-Aldrich, St. Louis, MO, USA) using an Octet-RED instrument (Pall ForteBio, Fremont, CA, USA) operating at 25°C. The purified VH antibodies were loaded onto the His1K biosensor at saturating concentrations along with a series of diluents in PBS (pH 7.4) for 600 s, and then the VH antibodies in PBST (pH 7.4, containing 0.1% BSA and 0.02% Tween 20) were added onto the biosensor for 300 s. The equilibrium dissociation constant (*K*_d_) was analyzed after 300 s of dissociation and calculated with software. The commercial anti-OVA antibody was labeled with biotin (Thermo Fisher Scientific, Rockford, IL, USA) and then loaded onto the SA biosensor for BLI detection.

### VH antibody ELISA and dot blotting

The microtiter plates were coated with 20 μg of an OVA solution (~100 μl) per well and blocked with a 2% skimmed milk-PBS solution. VH antibodies were added, the plates were incubated at room temperature for 1 h, washed five times with 0.1% PBST, and finally incubated with an HRP-conjugated anti-His antibody (ZSGB-BIO, Los Altos, CA, USA). The plates were then washed again with PBST and incubated with the TMB peroxidase substrate (BioLegend, San Diego, CA, USA). The absorbance was measured at 450 nm. The supernatant from IPTG-induced and uninduced *E. coli* strain BL21, the His6-tagged protein, an irrelevant protein containing the GFP-tag, and PBS served as controls for this experiment.

Antigens were immobilized on nitrocellulose membranes at 37°C for 10 min. The membrane was incubated with blocking solution (5% skimmed milk) for 1 h and then washed five times with TBST (Tris-buffered saline containing 0.1% Tween 20). The purified VH antibodies were pre-incubated with blocking solution at 4°C for 1 h and then applied to each well. After a 2 h incubation, the apparatus was washed five times with TBST and incubated with an HRP-conjugated anti-His antibody. After five additional washes with TBST, the membrane was treated with ECL-Plus reagent (Amersham Biosciences, Amersham, Buckinghamshire, UK) and detected using a CCD image analyser (LAS-3000, Fuji Photo Film Co. Ltd., Kanagawa, Japan). The supernatant from *E. coli* strain BL21, His6-tagged proteins and an irrelevant protein containing the GFP-tag served as controls for this experiment.

### Statistics

Each experiment was repeated at least three times. The results are presented as the means ± SEM. The statistical significance of differences between two groups of means was determined using the unpaired Student's *T*-test. The statistical significance of differences between three or more groups of means was determined using analysis of variance (ANOVA). *P* < 0.05 was considered statistically significant.

## Results

### Generation of a mouse line carrying the γ1 CH1 deletion

**Figure 1:**
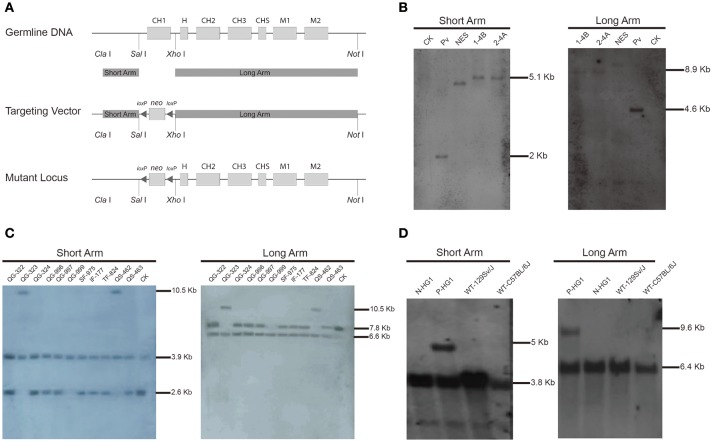
Construction of the targeting vector and generation of the γ1-CH1 deleted mice.**(A)** Targeting strategy for the genetic modification designed to delete the γ1 CH1 exon. **(B)** Identification of correctly targeted ES cell clones by Southern blotting. Southern blotting was performed using the DIG-labeled probe for the *neo* gene. Genomic DNA was digested with *Xho* I + *Eco*R I and *Sal* I + *Hin*d III to detect the short (5.05 kb) and long (8.94 kb) homologous arms, respectively. CK: 129/SvJ wild type mice; Pv: pPNIII vector digested with *Eco*R I; NES: negative ES cell clone 1–2A. **(C)** Identification of homozygous knockout mice by Southern blotting. Genomic DNA was digested with *Sph* I + *Dra* I to detect the homologous arms. The positive mice, QG-999, exhibited a 6.63 kb band for the long homologous arm and a 3.91 kb band for the short homologous arm. **(D)** Identification of HG1 mice with the *neo* gene deleted by Southern blotting. Genomic DNA was digested with *Sph* I + *Nco* I and *Sph* I + *Eco*N I to detect the short and long homologous arms, respectively. Positive mice exhibited a 5.06 kb band for the short homologous arm and a 9.44 kb band for the long homologous arm. P-mouse, positive mouse; N-mouse, negative mouse.

We initially aimed to generate a mouse line in which the CH1 exon of the γ1 gene (encoding IgG1) was substituted with a neomycin gene (*neo*). As shown in Figure 1A, a targeting vector based on the backbone of pPN III plasmid was constructed to contain a *neo* gene, which was flanked by two *lox*P sites and two arms homologous to the murine γ1 sequences. The construct was then transfected into mouse embryonic stem (ES) cells. After a standard procedure of positive and negative selection, putative targeted ES cell clones were isolated and subjected to further analysis by Southern blotting (Figure 1B). Two ES cell clones (1-4B and 2-4A) were finally confirmed to be the desired clones, in which the γ1 gene was correctly targeted.

ES cell clone 2-4A was injected into blastocysts to produce the F0 generation of chimeric mice, which were further crossbred with wild type (WT) mice to obtain F1 offspring. Then the F1 offspring were mated for homozygous mice, the offspring were genotyped by Southern blotting, which identified a homozygous individual (QG-999) in which both γ1 alleles were correctly targeted (Figure 1C). Unfortunately, no IgG1 antibodies were detected in the serum of the genetically modified homozygous mice, which was not too surprising based on previous reports that the presence of an antibiotic-resistance gene can impact the expression of adjacent endogenous genes (41, 42). Thus, we bred the QG-999 line with Tg (EIIa-cre) C5379 Lmgd, a mouse line that constitutively expresses Cre proteins, to delete any sequence flanked by *lox*P sites. We selected the offspring in which the *neo* gene was deleted for further breeding to establish a mouse line (hereafter called HG1). Homozygous HG1 mice exhibited a complete lack of the γ1 CH1 exon in both alleles, and the *neo* gene was also removed (Figure 1D).

### Expression of IgG1 in HG1 mice

**Figure 2:**
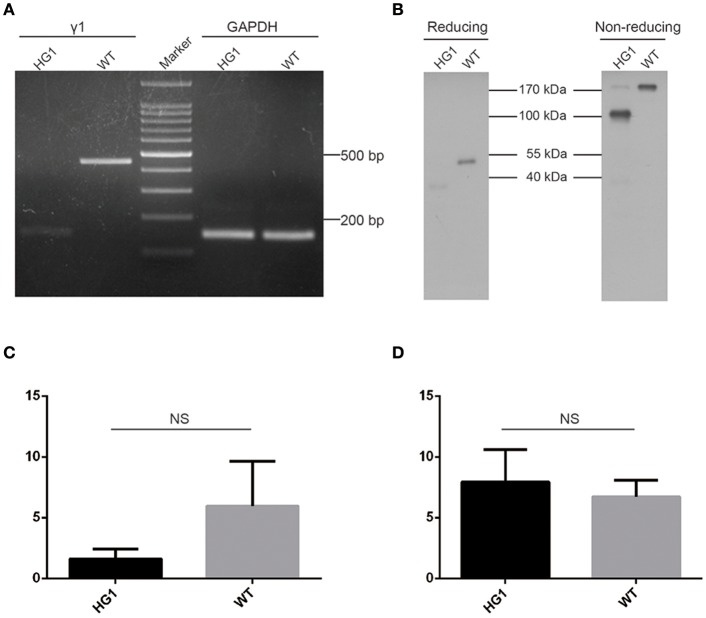
Characterization of γ1 gene expression in HG1 mice. **(A)** PCR detection of the γ1 transcript in HG1 mice. **(B)** Western blotting showing IgG1 levels in the serum of HG1 mice. **(C)** qRT-PCR analysis of the transcription of the γ1 gene in HG1 mice. **(D)** qRT-PCR analysis of the transcription of the μ gene in HG1 mice.

A sense primer derived from the heavy chain joining (JH) exon and an anti-sense primer derived from γ1 CH2 exon were used to perform RT-PCR to determine whether the JH exon had correctly spliced with the γ1 hinge exon. As expected, a PCR product was detected in HG1 mice that was ~300 bp shorter than the product amplified in WT mice (Figure 2A). Furthermore, Western blotting under reducing conditions revealed an ~40 kDa band for the IgG1 heavy chain protein that was ~10 kDa (corresponding to the molecular weight of the CH1 domain) smaller than the 50 kDa IgG1 heavy chain expressed in WT mice (Figure 2B). The shorter IgG1 heavy chain in HG1 mice formed dimers with a molecular weight that was ~70 kDa (the molecular weight of each light chain was ~25 kDa) less than the size of the dimers in WT mice, as shown by Western blotting under non-reducing conditions (Figure 2B), suggesting that no light chains were covalently associated with the heavy chain dimers.

Real-time quantitative PCR (qRT-PCR) was employed to quantitatively assess the transcription of the truncated IgG1 mRNA in HG1 mice using the IgM heavy chain as a control. The reduction of IgG1 expression in HG1 mice (~2.7-fold, *p* > 0.05) was observed compared with the WT mice (Figure 2C), whereas the transcriptional level of IgM heavy chain in HG1 mice was similar to WT mice (Figure 2D).

### IgG1 heavy chains were not covalently or non-covalently associated with light chains

**Figure 3:**
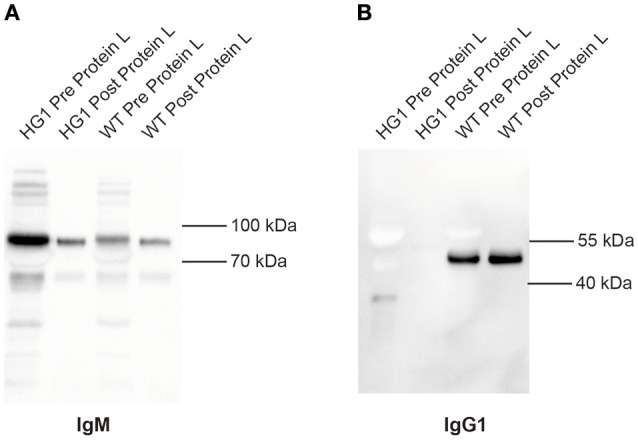
IgG1 does not associate with light chains in HG1 mice. **(A)** Western blotting indicating the levels of IgM derived from the sera of HG1 and WT mice before and after the protein L treatment. **(B)** Western blot showing the levels of IgG1 derived from the sera of HG1 and WT mice before and after the protein L treatment.

Although the aforementioned Western blotting results did not reveal a covalent association of light chains with the IgG1 heavy chains in HG1 mice, we could not exclude the possibility that the IgG1 heavy chain might be non-covalently associated with light chains. In murine serum, the κ and λ comprises about 95 and 5% of light chains, respectively. Protein L can specifically bind to any kappa chain-containing Ig classes. Protein L magnetic beads were used to pull down kappa chain- containing Ig classes from the sera of both WT and HG1 mice, and these purified immunoglobulins were further subjected to Western blotting analysis to further examine the potential non-covalent interaction. As shown in Figure 3A, as expected, IgM was consistently detected in serum samples from both WT and HG1 mice, regardless of the pre-purification with protein L beads. In contrast to IgG1 in wild type mice, IgG1 was not detected in the protein L-purified samples from HG1 mice, clearly suggesting that the IgG1 heavy chains were not associated with the κ light chains in HG1 mice (Figure 3B).

### The use of variable (V), diversity (D), and joining (J) gene segments and the length of CDR3 were not significantly different between the WT and HG1 mice

**Figure 4:**
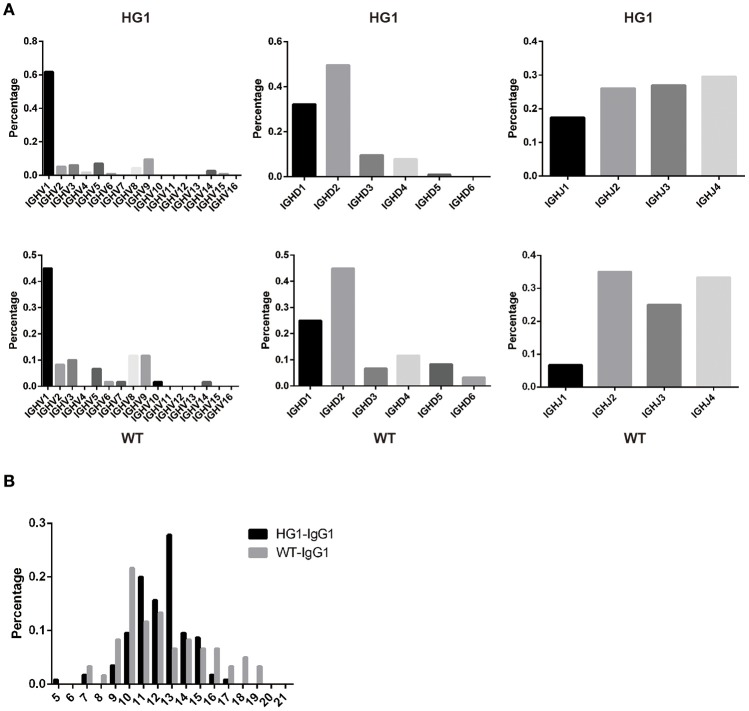
VH gene usage and CDR3 length analyses. **(A)** The usage of the V, D, and J genes of the γ1 transcripts in HG1 (upper panel) and WT (lower panel) mice. **(B)** Analysis of the length of CDR3 in the IgG1 heavy chains in HG1 and WT mice.

As IgG1 was expressed as a heavy chain-only antibody in HG1 mice, we were interested in determining whether the usage of V, D, and J segments of IgG1 in these mice was altered compared with IgG1 in WT mice. Thus, the expressed IgG1-VH fragments were amplified from the RNA obtained from the spleens of both HG1 and WT mice and sequenced. After removing a large number of redundant sequences, 115 and 60 VH sequences with unique CDR3 derived from HG1 and WT mice, respectively, were obtained and analyzed. Frequency analyses of the usage of the specific V, D, and J segments in these clones revealed no marked differences, except that the usage of the VH1 family was increased in HG1 mice (62 vs. 45%; Figure 4A).

Further analyses of the CDR3 length in IgG1 heavy chains in both HG1 and WT mice did not reveal significant differences, as the average length was 12.2 ± 1.92 aa in HG1 mice and 12.3 ± 3.03 aa in WT mice (Figure 4B). Consistent with these findings, the IgM CDR3 length was 11.3 ± 2.89 aa in HG1 mice and 11.9 ± 2.79 aa in WT mice, exhibiting a very similar length pattern (data not shown).

### The IgG1 expression level was significantly reduced in HG1 mice

**Figure 5:**
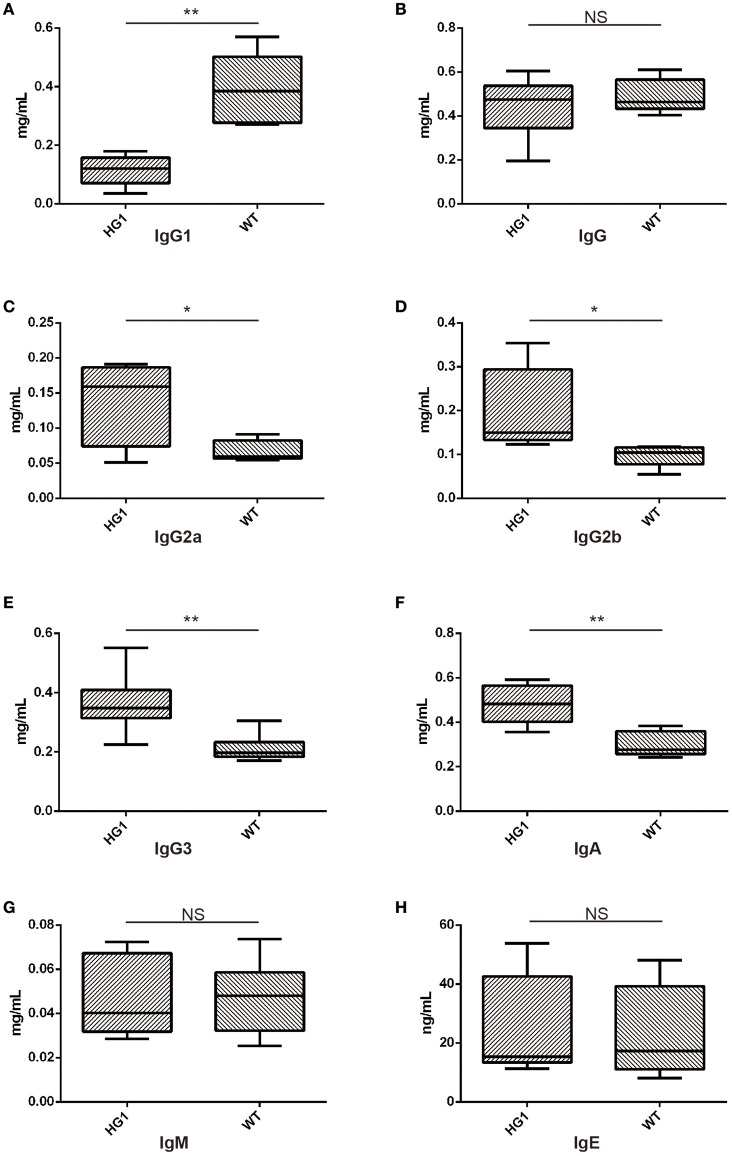
ELISA detection of Ig concentrations in the sera of HG1 and WT mice. (**A–H**) Concentrations of IgG1, IgG, IgG2a, IgG2b, IgG3, IgA, IgM, and IgE, respectively. The asterisk rating system is used to indicate the *P* value: *P < 0.05, **P < 0.01, ***P < 0.001 and NS stands for Not Statistically Significant.

We measured the concentrations of all Ig classes by ELISA in HG1 mice and WT mice to examine whether the genetic removal of the γ1 CH1 exon affected the levels of Ig classes in HG1 mice. As shown in Figure 5, consistent with the real-time PCR data, the IgG1 concentration was significantly reduced in the sera of HG1 mice (Figure 5A), although the total IgG concentration in these mice was similar to WT mice (Figure 5B). Perhaps compensating for the reduced IgG1 level in HG1 mice, the concentrations of IgG2a, IgG2b, IgG3, and IgA were significantly increased (Figures 5C–F, respectively), whereas the levels of IgM and IgE in these mice were not significantly altered compared with those in WT mice (Figures 5G,H).

### HG1 mice exhibited a deficient antigen-specific IgG1 response

**Figure 6:**
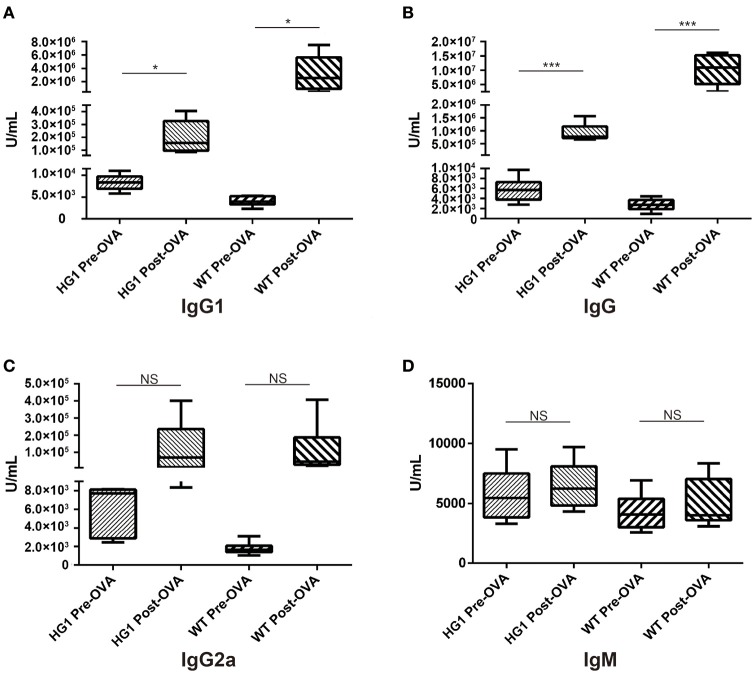
ELISA detection of OVA-specific Ig concentrations in the sera of HG1 and WT mice before and after OVA immunization. **(A–D)** Titres of IgG1, IgG, IgG2a, and IgM, respectively. The asterisk rating system is used to indicate the *P* value: *P < 0.05, **P < 0.01, ***P < 0.001 and NS stands for Not Statistically Significant.

Both groups of mice were immunized with OVA to determine whether or how the humoral response was altered in HG1 mice compared to that in WT mice. Serum samples were collected to determine the concentrations of OVA-specific IgG1, total IgG, IgM, and IgG2a both before and after immunization. While the titre of OVA-specific IgG1 was significantly increased in WT mice after immunization (nearly 800-fold), the value was only slightly increased in HG1 mice (only ~16-fold; Figure 6A). Similarly, the OVA-specific IgG titre was increased ~3,704-fold in the WT group but only 140-fold in HG1 mice after immunization (Figure 6B). However, no significant differences in the IgG2a and IgM responses were observed between these two groups of mice upon immunization (Figures 6C,D). Immunization with several other antigens also showed similar IgG1 and total IgG responses, suggesting that HG1 mice exhibited a deficient IgG1 response upon immunization (data not shown).

### VH phage library construction and antigen-specific VH panning

Although HG1 mice appeared to have a substantially decreased IgG1 response compared with WT mice, a certain level of a specific IgG1 response was still observed following OVA immunization. This observation encouraged us to investigate whether OVA-specific VH could be isolated using the HG1 mice. To test this, we extracted total splenic RNA from the OVA-immunized HG1 mice and employed it as the template to amplify IgG1-associated VH sequences using nested PCR with a set of upstream primers designed to cover all known VH families. All amplified VH sequences were cloned into the phagemid vector pHEN-2 and further used to transform the *E. coli* strain TG1 to generate a VH library of ~1.0 × 10^8^ cfu (colony forming units). The transformed bacterial library was then infected with M13 helper phage to generate a phage library.

Bio-panning of the phage library was then performed in 96-well plates coated with OVA using a standard procedure (43). After three rounds of panning, the outputs were enriched, and 47 clones were selected (Figure S1A). The clones were further characterized by sequencing, and only 12 unique sequences were observed. As shown in Figure S1B, the majority of VHs, with the exception of 3A-42, exhibited high sequence identities.

### Selected VH domains specifically recognized OVA

**Figure 7:**
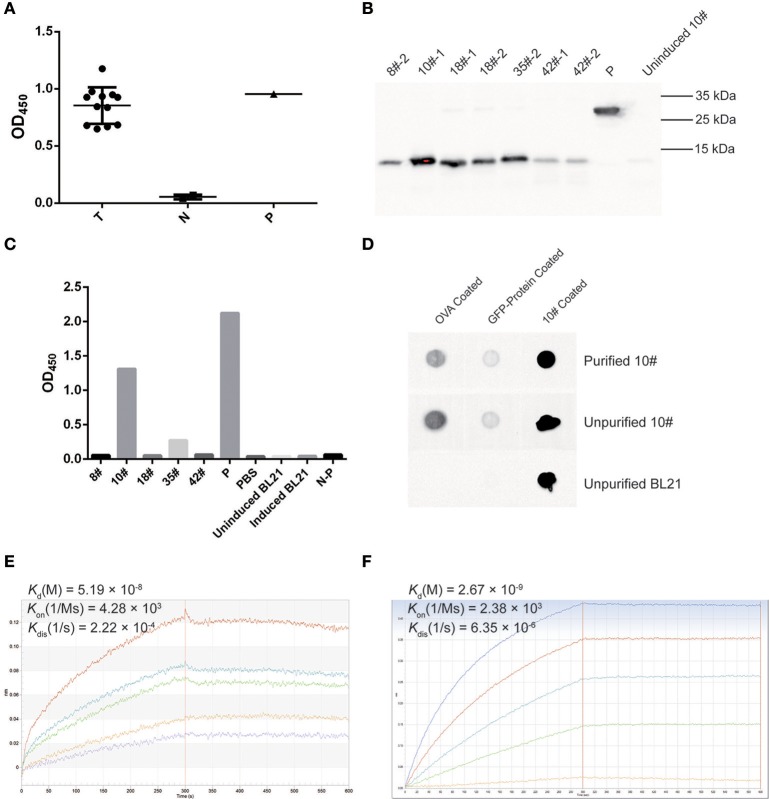
OVA-specific nano-body screening and affinity evaluation. **(A)** The binding capability of OVA-specific phages was evaluated by phage ELISA using an anti-M13-HRP antibody. A well coated with M13 was used as a positive control (P), an irrelevant protein containing the GFP-tag, PBS, and M13 served as negative controls (N). Twelve screened phages were tested as samples (T). The OD_450_ values are indicated. **(B)** Western blotting for OVA-specific nano-bodies. A His6-tagged protein was used as a control (P), and the un-induced culture product of #10 clone was used as a negative control. **(C)** The binding of expressed OVA-specific nano-bodies was evaluated by ELISA. A well coated with His6-tagged protein was used as a positive control (P), and an irrelevant protein containing the GFP-tag (N-P), PBS, IPTG-induced and uninduced BL21 culture product were used as negative controls. The OD_450_ values are indicated. **(D)** Dot blotting of expressed OVA-specific nano-body #10. An irrelevant protein containing the GFP-tag was used as a control. **(E)** Binding kinetics of the #10 nano-body (20 μg/ml) to OVA (2.22, 1.11, 0.56, 0.28, 0.14 μM) were detected using the Octet-RED instrument. The equilibrium dissociation constant (*K*_d_) is indicated. **(F)** Binding kinetics of a commercial anti-ovalbumin antibody (5 μg/ml) to OVA (3.84, 1.92, 0.96, 0.48, 0.24 μM) were detected using the Octet-RED instrument.

A phage ELISA was conducted to evaluate whether selected VHs specifically recognized OVA, and the results showed that all 12 selected clones specifically bound to OVA (Figure 7A). Based on the sequence identities and ELISA results, five VHs (#8, #10, #18, #35, and #42) were chosen for expression in a prokaryotic expression system. The selected VHs were amplified and ligated into the pET-28a (+) vector, in which a His6-tag was fused at the C-terminus of the VHs for further purification. These vectors were transformed into *E. coli* strain BL21, and the supernatants were subsequently collected to detect the expression of VH antibodies by Western blotting using an anti-His antibody. As all five VHs were successfully expressed (Figure 7B), an ELISA was further employed to confirm whether these antibodies specifically bound OVA. As shown in Figure 7C, two VH antibodies (#10 and #35) possessed the ability to bind the OVA antigen.

Subsequently, the expressed #10 and #35 VHs were purified using Ni-NTA His•Bind resin, and these two purified VH antibodies were both detected as monomers after electrophoresis on non-reducing polyacrylamide gels (data not shown). An ELISA was further employed to detect the binding affinity of the purified VH antibodies, for which an irrelevant protein containing the His6-tag was considered as a control. The #10 and #35 VH antibodies exhibited sufficient affinity for purified OVA (data not shown). However, compared with the controls, the #10 VH antibody showed a better binding capacity, and therefore this antibody was used in subsequent analyses. Dot blotting with purified OVA-coating was then employed to verify the binding affinity of the #10 VH antibody, and a coating with an irrelevant protein containing the GFP-tag or purified #10 VH antibody was used as control. The #10 VH antibody displayed better recognition and binding to purified OVA, and the #10 VH antibody showed low non-specific binding affinity for the irrelevant protein containing the GFP-tag (Figure 7D).

Finally, the affinity of the #10 VH antibody was assessed with biolayer interferometry (BLI) using Octet-RED. We analyzed the interaction of the #10 VH antibody with purified OVA. The #10 VH antibody interacted with purified OVA with high affinity, displaying an equilibrium dissociation constant (*K*_d_) of 51.9 nM (Figure 7E). In contrast, the equilibrium dissociation constant (*K*_d_) of the anti-ovalbumin antibody (purchased from Abcam) for OVA was 2.67 nM, which was also detected by Octet-RED (Figure 7F).

## Discussion

Naturally, HcAbs, which show great potential in many applications such as laboratory practice, analysis of small chemicals, clinical diagnosis, and therapeutic applications (17, 44–49), are found in camelids and sharks. In this study, we set out to investigate whether the precise genetic removal of the CH1 exon from an IgG-encoding gene would enable the production of functional HcAbs in mice. Using gene targeting technology, we generated a mouse line in which the γ1 CH1 exon was deleted, and although these mice expressed heavy chain-only IgG1, they mounted only a weak IgG1-specific response when immunized with particular antigens. We were able to isolate antigen-specific single VH domain antibodies from these mice, although these antibodies exhibited a lower antigen binding affinity than conventional monoclonal antibodies. Therefore, this study reveals the possibility of using genetically modified small laboratory animals to produce monoclonal single VH domain antibodies. Attempts to produce heavy chain only antibodies in mice have previously been reported. For example, using μMT mice, Janssens et al. have generated transgenic mice containing hybrid chimeric loci, where non-rearranged llama VHH exons were linked with CH1 exon-removed human IgH constant region genes (50). These mice were shown to be able to produce chimeric llama-human heavy chain only antibodies. In this study, we set out to investigate whether fully murine heavy chain only antibodies could be produced if we remove the CH1 exon of endogenous mouse γ1 constant region gene precisely via gene targeting.

An interesting observation in this study was that the deletion of γ1 CH1 exon led to a reduced IgG1 production in the HG1 mice. Theoretically, there are several possibilities behind the altered phenotype. First, the deletion of γ1 CH1 exon may somehow down-regulate the level of γ1 germline transcription, which would consequently decrease class switching efficiency of IgM^+^ B cells to IgG1^+^ B cells. Second, when the γ1 CH1 was deleted, the recombined VDJ exon would have to be spliced onto the hinge exon during RNA processing. The deletion of γ1 CH1 exon may also influence the splicing efficiency of the modified primary γ1 heavy chain transcripts. This would also change the expression of IgG1 even the class switching process was not affected. Finally, even if the IgG1 heavy chain could be successfully expressed in some B cells, there is still a possibility that the expressed IgG1 heavy chain could not form a functional B cell receptor, and thus, these B cells would be eliminated by apoptosis.

Based on the genetically modified mice generated here from which the data were derived, we can learn some important lessons to improve strategies for the production of single VH domain antibodies using small laboratory animals. In HG1 mice, deletion of the γ1 CH1 exon apparently decreased IgG1 expression, but increased the levels of other IgG subclasses. The increased levels of other IgG subclasses might compensate for the decreased IgG1 expression. Two strategies could be adopted to increase the production of heavy chain-only antibodies in the genetically modified HG1 mice. The first is to remove the CH1 exons from all other IgG subclasses, and the other alternative is to disrupt all other IgG subclass-encoding genes and maintain only the CH1-deleted γ1 gene in the IgH locus. Similar to the second idea, we could also use other laboratory animals, such as rabbits, to generate the same genetic modification, as rabbits have only one IgG-encoding gene in their genome.

Another major issue that must be considered is that HG1 mice appeared to be largely deficient in a specific IgG1 response to particular antigens, which is likely the key explanation for our failure to isolate single VH domain antibodies with high affinity for specific antigens. Indeed, as compensation for the lack of light chains, the natural camelid VHH antibody usually contains changes in specific residues that are encoded in the camelid germline IgH locus and long CDR3 sequence. An analysis of VH sequences of IgG1 in HG1 mice did not show either changes in specific residues or an increased CDR3 length. The IgG1 antibodies were likely hypofunctional in HG1 mice, although these antibodies were expressed and secreted into the serum. If this hypothesis is valid, the integration of camelid VHH gene segments into the VH gene locus of HG1 mice may enhance the production of functional HcAbs in these mice.

In summary, although we obtained the single domain VH antibodies with lower affinity than expected using the HG1 mice, this study, to some extent, reveals the possibility of producing monoclonal HcAbs using mice bearing appropriate genetic modifications.

## Author contributions

YZ, SY, and XY designed the experiments. YZ, XQC, and TZ wrote the manuscript. TZ, XQC, and DY performed the main experimental works and contributed equally. TZ, FL, NH, XC, and JW performed the animal gene modification. XQC, DY, SH, LM, YF, and YM performed the IgG1-HcAb detection. XQC and DY performed the animal immunization, antigen-specific nano-antibodies selection and detection. LR and HH provided materials and technical help. YZ, TZ, and XQC revised and completed the final draft of the article. All authors approved the submitted version.

### Conflict of interest statement

The authors declare that the research was conducted in the absence of any commercial or financial relationships that could be construed as a potential conflict of interest. The reviewer PB and handling editor declared their shared affiliation at the time of the review.
