# Corrigendum: Genetic Removal of the CH1 Exon Enables the Production of Heavy Chain-Only IgG in Mice

**DOI:** 10.3389/fimmu.2019.00398

**Published:** 2019-03-08

**Authors:** Tianyi Zhang, Xueqian Cheng, Di Yu, Fuyu Lin, Ning Hou, Xuan Cheng, Shanshan Hao, Jingjing Wei, Li Ma, Yanbin Fu, Yonghe Ma, Liming Ren, Haitang Han, Shuyang Yu, Xiao Yang, Yaofeng Zhao

**Affiliations:** ^1^State Key Laboratory of Agrobiotechnology, College of Biological Sciences, National Engineering Laboratory for Animal Breeding, China Agricultural University, Beijing, China; ^2^State Key Laboratory of Proteomics, Beijing Proteome Research Center, National Center for Protein Sciences (Beijing), Beijing Institute of Lifeomics, Beijing, China

**Keywords:** HcAbs, nanobody, CH1 domain, mouse, phage display, single domain antibodies

In the original article “Janssens R, et al., Generation of heavy-chain-only antibodies in mice. Proc Natl Acad Sci USA. (2006) 103:15130–35” was not cited. The citation has now been inserted in the **Discussion**, paragraph one, and should read:

“Naturally, HcAbs, which show great potential in many applications such as laboratory practice, analysis of small chemicals, clinical diagnosis, and therapeutic applications (17, 44–49), are found in camelids and sharks. In this study, we set out to investigate whether the precise genetic removal of the CH1 exon from an IgG-encoding gene would enable the production of functional HcAbs in mice. Using gene targeting technology, we generated a mouse line in which the γ1 CH1 exon was deleted, and although these mice expressed heavy chain-only IgG1, they mounted only a weak IgG1-specific response when immunized with particular antigens. We were able to isolate antigen-specific single VH domain antibodies from these mice, although these antibodies exhibited a lower antigen binding affinity than conventional monoclonal antibodies. Therefore, this study reveals the possibility of using genetically modified small laboratory animals to produce monoclonal single VH domain antibodies. Attempts to produce heavy chain only antibodies in mice have previously been reported. For example, using μMT mice, Janssens et al. have generated transgenic mice containing hybrid chimeric loci, where non-rearranged llama VHH exons were linked with CH1 exon-removed human IgH constant region genes (50). These mice were shown to be able to produce chimeric llama-human heavy chain only antibodies. In this study, we set out to investigate whether fully murine heavy chain only antibodies could be produced if we remove the CH1 exon of endogenous mouse γ1 constant region gene precisely via gene targeting.”

The authors apologize for this error and state that this does not change the scientific conclusions of the article in any way. The original article has been updated.

## References

[50] Janssens R Dekker S Hendriks RW Panayotou G van Remoortere A San JK Generation of heavy-chain-only antibodies in mice. Proc Natl Acad Sci USA. (2006) 103:15130–35. 10.1073/pnas.0601108103 17015837 PMC1586177

