# Supplementary material for: Genetic Removal of the CH1 Exon Enables the Production of Heavy Chain-Only IgG in Mice
Source: Front Immunol. 2018 Sep 25;9:2202. doi: 10.3389/fimmu.2018.02202 (PMC6167435; doi:10.3389/fimmu.2018.02202)
Supplement: Supplementary file 1 [file Data_Sheet_1.docx]

**Genetic Removal of the CH1 Exon Enables the Production of Heavy Chain-only IgG in Mice**

*Tianyi Zhang ^1^†‡, Xueqian Cheng ^1^‡, Di Yu ^1^‡, Fuyu Lin ^2^, Ning Hou ^2^, Xuan Cheng ^2^, Shanshan Hao ^1^, Jingjing Wei ^1^, Li Ma ^1^, Yanbin Fu ^1^, Yonghe Ma ^1^, Liming Ren ^1^, Haitang Han ^1^, Shuyang Yu ^1*^, Xiao Yang ^2*^, and Yaofeng Zhao ^1*^*

*** Correspondence:**

**Yaofeng Zhao**

[yaofengzhao@cau.edu.cn](mailto:yaofengzhao@cau.edu.cn)

**Xiao Yang**

[yangx@bmi.ac.cn](mailto:yangx@bmi.ac.cn)

**Shuyang Yu**

[ysy@cau.edu.cn](mailto:ysy@cau.edu.cn)

**
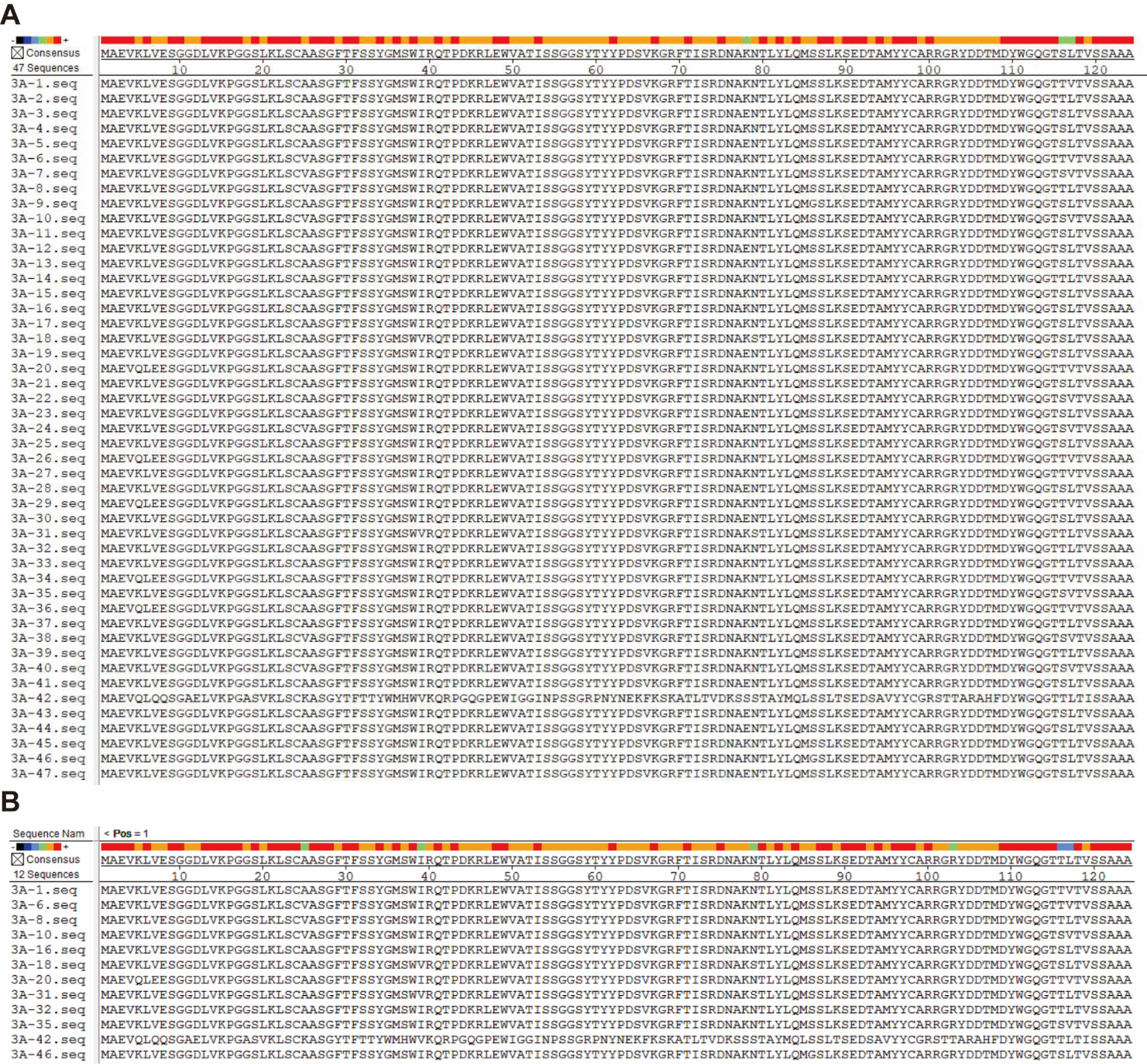
SUPPLEMENTARY FIGURE 1**

**FIGURE S1.** Amino acid sequences of selected clones after bio-panning. (**A**) Amino acid sequences of selected 47 clones after three rounds of bio-panning. (**B**) The 12 unique amino acid sequences obtained after three rounds of bio-panning are indicated.
